# Kupffer Cells Promote the Differentiation of Adult Liver Hematopoietic Stem and Progenitor Cells into Lymphocytes via ICAM-1 and LFA-1 Interaction

**DOI:** 10.1155/2019/4848279

**Published:** 2019-07-01

**Authors:** Deping Meng, Yuhong Qin, Nan Lu, Keke Fang, Yuan Hu, Zhigang Tian, Cai Zhang

**Affiliations:** ^1^Institute of Immunopharmacology and Immunotherapy, School of Pharmaceutical Sciences, Shandong University, Jinan, 250012 Shandong, China; ^2^Institute of Diagnostics, School of Medicine, Shandong University, Jinan, 250012 Shandong, China; ^3^Institute of Immunology, School of Life Sciences, University of Science and Technology of China, Hefei, 230027 Anhui, China

## Abstract

It has been reported that the adult liver contains hematopoietic stem and progenitor cells (HSPCs), which are associated with long-term hematopoietic reconstitution activity. Hepatic hematopoiesis plays an important role in the generation of cells involved in liver diseases. However, how the progenitors differentiate into functional myeloid cells and lymphocytes in the liver microenvironment remains unknown. In the present study, HSPC transplantation experiments were used to confirm that adult murine liver HSPCs differentiate into both myeloid cells and lymphocytes (preferentially T cells) compared with bone marrow HSPCs. Using a coculture system comprised of kupffer cells and HSPCs, we found that kupffer cells promote adult liver HSPCs to primarily generate T cells and B cells. We then demonstrated that kupffer cells can also promote HSPC expansion. A blockade of intercellular cell adhesion molecule-1 (ICAM-1) in a liver HSPC and kupffer cell coculture system impaired the adhesion, expansion, and differentiation of HSPCs. These results suggest a critical role of kupffer cells in the maintenance and promotion of adult mouse liver hematopoiesis. These findings provide important insight into understanding liver extramedullary hematopoiesis and its significance, particularly under the state of some liver diseases, such as hepatitis, nonalcoholic fatty liver disease (NAFLD), and hepatocellular carcinoma (HCC).

## 1. Introduction

It has been established that the liver is the major hematopoietic organ during fetal period. After birth, hematopoietic stem cells reside primarily in the bone marrow. In adults, extramedullary hematopoiesis occurs in the liver, spleen, and other solid organs when hematopoiesis in the bone marrow fails, as a result of some pathological conditions [1–4]. It has been reported that the adult liver contains Lin^lo/-^sca-1^+^c-kit^+^ cells which exhibit colony-forming ability *in vitro* and reconstruct the multilineage hematopoiesis of lethally irradiated recipient mice *in vivo* [3]. Later, CD45^+^ liver side population (SP) cells, isolated based on Hoechst 33342 dye staining, are reported which have the potential of hematopoietic reconstitution and generate the lymphoid, myeloid, and erythroid lineages in the lethally irradiated recipient mice [4]. Moreover, HSPCs were found in the adult human liver, and liver grafts after extensive perfusion can restore the recipient multilineage hematopoiesis to some extent [5–7]. Although hepatic hematopoiesis plays an important role in the generation of cells involved in tumor surveillance and rejection [8], there is a lack of systemic research comparing the differences between hematopoiesis and lymphogenesis between the adult liver and bone marrow and how the liver microenvironment contributes to these events. The quiescence, proliferation, and differentiation of HSPCs in the bone marrow require a specific niche. Macrophages, endothelial cells, perivascular cells, and other stromal cells play critical roles in maintaining the hematopoietic stem cell pool and regulating HSPC activity by producing a wide variety of cytokines, hematopoietic growth factors, chemokines, and adhesion molecules [9–11]. Among these, adhesion receptors and their ligands (e.g., ICAM-1/LFA-1 and VCAM-1/VLA-4) are important for regulating hematopoietic function and anchoring HSPCs to the niche [12, 13]. Indeed, an ICAM-1 deficiency impairs the quiescence and repopulation activity of HSPCs in the bone marrow niche [13, 14]. However, factors in the adult liver hematopoietic niche for HSPCs remain poorly understood.

In the present study, we detected the presence of heterogeneous Lin^−^Sca-1^+^c-Kit^+^ (LSK, contains hematopoietic stem cells and multipotent progenitors) cells [15] in the adult murine liver. Through HSPC transplantation experiments, we observed that liver LSK cells differentiate into both myeloid cells and lymphocytes, particularly preferentially generated T cells compared with bone marrow HSPCs. We next explored how the liver microenvironment promotes liver hematopoiesis and lymphocyte differentiation and which factors are required. We found that kupffer cells could induce liver HSPCs to differentiate into a relatively high proportion of T and B lymphocytes in an ICAM-1/LFA-1 interaction-dependent manner.

## 2. Materials and Methods

### 2.1. Animal Strains and Treatment Protocol

Six- to eight-week-old male C57BL/6j mice were obtained from Hua Fukang Biological Technology Co. Ltd. (Beijing, China) and maintained in a pathogen-free animal facility. Male and female C57BL/6-Ly5.1 (CD45.1) were obtained from Beijing Vital River Laboratory Animal Technology Co. Ltd. An adult murine liver extramedullary hematopoietic model was established by an intraperitoneal injection of 10 *μ*g/mL LPS for three days. An intravenous injection of clodronate-liposome (CL, Shanghai Yisheng Biological Technology Co. Ltd.) was used to remove the liver kupffer cells [16]. CL was injected to mice on the first and third days. From the second day, C57BL/6j mice were treated with an intraperitoneal injection of 10 *μ*g/mL LPS for three days. The tissue was harvested on the fourth day and tissue mononuclear cells were detected by flow cytometry. All animal protocols were approved by the Institutional Animal Care and Use Committee at Shandong University.

### 2.2. Colony-Forming Unit Assay

A total of 1 × 10^3^ sorted bone marrow or liver LSK cells were suspended in methyl cellulose semisolid medium (1.5% methylcellulose in IMDM) and seeded into a 24-well culture plate to which hematopoietic growth factors (SCF 50 ng/mL, Flt3-L 50 ng/mL, IL-3 20 ng/mL, IL-7 20 ng/mL, M-CSF 50 ng/mL, and GM-CSF 50 ng/mL) were added. The cells were incubated at 37°C in 5% CO_2_ for 14 days. The number of colonies (with >50 cells) of colony-forming unit-granulo-macrophage (CFU-GM) and CFU-macrophage (CFU-M) was counted under a light microscope on day 14 [17].

### 2.3. Flow Cytometry

Bone marrow and liver mononuclear cells were harvested [18]. Single cell suspensions were blocked with an Fc receptor CD16/CD32 at room temperature. After 10 min, the cells were stained with a cocktail of antibodies. For HSC staining, mononuclear cells from the bone marrow and liver were stained with antibodies against the lineage antibodies cocktain-percpcyTM5.5, sca-1-APC, CD117-PE-eFluor610, Flk2-PE, and CD34-FITC cocktail. After 30 min of staining at room temperature, the cells were washed with a 1× phosphate buffer solution (PBS). For the transplantation studies, peripheral blood was obtained by retro-orbital bleeding and red blood cell depletion. Samples were stained for 30 min at room temperature with antibodies against CD45.1-PE-CF594, CD45.2-APC, CD3-PE-cy7, CD19-PE, CD11b-FITC, and NK1.1-APC-cy7. For the coculture studies, samples were stained for 30 min at room temperature with antibodies against CD3-PE-cy7, CD19-APC-cy7, CD11b-PE, and NK1.1-APC. For flow cytometric sorting and LSK cell analysis, liver mononuclear cells were stained with antibodies against lineage antibody cocktain-percpcyTM5.5, sca-1-APC, CD117-PE-eFluor610, LFA-1-PE-cy7, and VLA-4-FITC. Kupffer cells were stained with antibodies against F4/80-PE-CF594, CD11b-PE, ICAM-1-APC, and VCAM-1-FITC. For Ki-67 staining of LSK cells, the samples were stained for 30 min at room temperature with antibodies against lineage antibody cocktain-percpcyTM5.5, sca-1-APC, CD117-PE-eFluor610, and Ki-67-PE. The cells were analyzed and sorted using a FACSAria III cell sorter (BD). The purity of sorting was higher than 95%. The antibodies are presented in Table S1.

### 2.4. Kupffer Cell Isolation

Kupffer cells were isolated by collagenase digestion and Percoll density gradient centrifugation. The mice were anaesthetized with 2 mg/mL/20 kg lidocaine prior to a laparotomy. The portal vein was cannulated with a 24 G indwelling needle, and the liver was perfused with 1-3 mL EGTA/HBSS solution. The inferior vena cava was rapidly cut off after the liver turned completely pale. Next, the liver was perfused with 37°C prewarmed collagenase solution for 5 min. The liver was then excised and transferred to a culture dish containing a collagenase solution and digested for 15 min in a 37°C incubator. The liver homogenate was then filtered to a 50 mL centrifuge tube. The cell suspension was centrifuged three times at 50 × g for 3 min. The final cell supernatant was centrifuged at 500 × g for 8 min. The cell precipitate was resuspended in 25% Percoll and slowly added to 50% Percoll. The 25%/50% Percoll gradient was centrifuged at 800 × g for 15 min. The interface of the gradient kupffer cell-enriched fraction was resuspended in 1× PBS and centrifuged at 500 × g for 8 min. The cellular precipitate was resuspended in 1 mL DMEM+10% FBS medium. The cells were then seeded into 24-well plates at a density of 1 × 10^5^ cells/well. The cells were incubated at 37°C in a 5% CO_2_ incubator. After 30 min, the medium was gently removed, and the cells were washed three times with 1× PBS, then replaced with 500 *μ*L fresh DMEM medium.

### 2.5. Real-Time PCR

The total RNA from the liver tissue was extracted using TRIZOL reagent (Invitrogen, Carlsbad, CA, USA). The RNA concentration was quantified using a Nanodrop 2000 (BioTek, Vermont, USA). cDNA was generated using a FastQuant RT Kit (Tiangen Biotech Co. Ltd., Beijing). Real-time polymerase chain reaction (qRT-PCR) was performed using a SYBR Green Supermix (Roche, Basel, Switzerland). The primers are presented in Table S2.

### 2.6. HSPC Adherence Assay

The HSPC adherence assay was performed according to the method described by Wang et al. [19] with certain modifications. Liver mononuclear cells were labeled with CFSE for 15 min then washed with 1× PBS. The cell suspensions were Fc blocked with anti-CD16/CD32 at room temperature. After 10 min, the cells were stained with a cocktail of lineage antibody cocktain-percpcy5.5, Sca-1-APC, and CD117-PE-eFluor610 antibodies. Next, CFSE^+^ LSK cells were sorted by flow cytometry. A density of 1 × 10^4^ sorted CFSE^+^ LSK cells in 500 *μ*L IMDM supplemented with 10% FBS per well was seeded to 1 × 10^5^ kupffer cell monolayer (incubated for 1 h in advance with 10 *μ*g/mL anti-ICAM-1 blocking antibodies) in a 24-well plate and incubated at 37°C. After 12 h, the plate was shaken for 30 s on the rocking bed at 120 rpm. Nonadherent CFSE^+^ cells were removed by pipetting. The cells were gently washed twice and counted by flow cytometry. The ratios of adherent CFSE^+^ LSK cells to those initially added were calculated.

### 2.7. Cytokine Detection by ELISA

Freshly isolated kupffer cells (1 × 10^5^ per well) were seeded into 24-well plates and incubated at 37°C. The culture supernatants were collected at 24 h and 48 h, respectively. The coculture supernatants were collected at 72 h. The level of IL-3, IL-6, SCF, TNF-*α*, IL-18, and IL-1*β* in cell culture supernatants was detected using ELISA kits (PeproTech, New Jersey, USA) in accordance with the manufacturers' instructions.

### 2.8. HSPC Transplantation

CD45.1 mice were lethally irradiated with a dose of 10 Gy. Mice were fed with water supplemented with 2 mg/mL neomycin. A total of 2 × 10^4^ LSK cells obtained from CD45.2 mice were mixed with 2 × 10^5^ unfractionated CD45.1^+^ competitor bone marrow cells and intravenously injected into irradiated CD45.2 recipient mice. Peripheral blood was obtained weekly and the proportion of lymphocytes and myeloid cells was calculated by flow cytometry.

### 2.9. Immunofluorescence Microscopy

Liver mononuclear cells were harvested and labeled with CFSE. CFSE^+^ LSK cells were sorted by flow cytometry and injected into mice via the tail vein. The next day, the liver tissue was harvested and soaked in OTC entrapment agent. Frozen sections of the liver tissue were made by first fixing the livers in 4% paraformaldehyde for 15 min. The samples were washed three times with 1× PBS for 5 min. Then, the tissues were incubated in 5% BSA for 30 min at room temperature [20] before the tissue sections were stained with anti-ICAM-1 and anti-F4/80 antibodies overnight at 4°C. The samples were then washed three times with 1× PBS for 5 min and stained with 7-amino-4-methylcoumarin-3-acetic acid (AMCA) goat anti-mouse IgG (H+L) and Alexa Flour 594 goat anti-rabbit IgG (H+L) at room temperature for 1 h. The samples were washed three times with 1× PBS for 5 min and microscopic images were acquired using a laser confocal microscope (LSM780, Carle Zeiss AG, Germany).

### 2.10. Statistical Analysis

The data were analyzed using GraphPad Prism 5 software (GraphPad Software Inc. USA). Statistical differences were calculated using a two-tailed Student *t*-test. A threshold value of *P* < 0.05 was considered significant.

## 3. Results

### 3.1. The Adult Murine Liver Contains Hematopoietic Progenitor Cells

**Figure 1:**
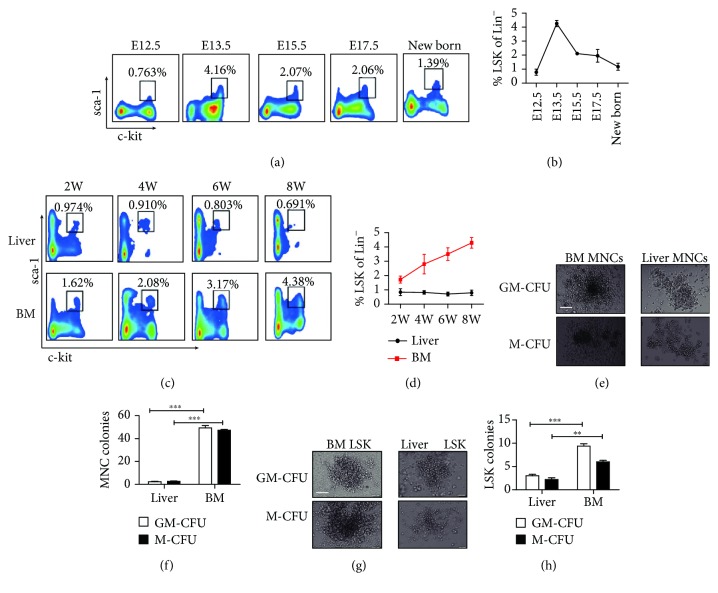
Detection of LSK cells in the different developmental stages of the liver and bone marrow. (a) The proportion of c-kit^+^ sca-1^+^ cells in the flow plot is gated from the fetal liver and newborn liver Lin^−^ cells (*n* = 3‐6). (b) Statistical analysis for the percentage of LSK cells gated from the Lin^−^ cells in the different developmental stages of the liver. (c) Flow cytometric plots show the percentage of LSKs among the Lin^−^ cells from the bone marrow or liver of young and adult mice (*n* = 3‐6). (d) Statistical analysis for the percentage of LSK cells gated from the Lin^−^ cells in the different developmental stages of the liver and BM. (e, g) Hematopoietic colony formation of the mononuclear cells (e) or LSK cells (g) from the adult liver or bone marrow (the picture shows a single colony in a well of 24-well cell culture plate). A total of 1 × 10^5^ bone morrow or liver mononuclear cells was freshly isolated from adult C57BL/6j mice, and 5 × 10^2^ bone marrow or liver LSK cells were sorted by FACS and plated into complete methylcellulose medium and incubated for 10 to 14 days. The number of colonies (≧50 cells are defined as one clone) was counted under an inverted phase contrast microscope. GM-CFU: the added cytokines included SCF, IL-3, FLT-3-L, IL-7, and GM-CSF. M-CFU: the added cytokines included SCF, IL-3, FLT-3-L, IL-7, and M-CSF. Picture original magnification: ×20 (e, g). Bar: 200 *μ*m. (f, h) Statistical analysis for the number of GM-CFU and M-CFU from MNCs or LSK cells of BM and liver (*n* = 3). All colonies were counted in a well of 24-well cell culture plate. Bars represent the mean ± SEM of three independent experiments. ^∗∗^ *P* < 0.01; ^∗∗∗^ *P* < 0.001.

Hematopoietic stem cells first appear in the aorta-gonad-mesonephros (AGM) region on embryonic day (E) 10.5, then migrate to the fetal liver around E11.5, where they undergo dramatic expansion [21–26]. Moreover, previous studies have identified the presence of LSK cells in the adult liver [3]. To confirm the presence of hematopoietic progenitor cells in the adult murine liver, we detected and compared the proportion of LSK cells in the livers from mice at different developmental stages by flow cytometry (Figure S1(a) and Figures 1(a)–1(d)). The results showed that there was a higher proportion of LSK cells in the fetal liver around E13.5 (Figures 1(a) and 1(b)) and subsequently decreased gradually from E13.5 to neonatal birth with a frequency of about 4.260% ± 0.227% to 1.320% ± 0.099% Lin^−^ cells (Figure 1(b)). From neonatal birth to adult, a small number of LSK cells remained in the liver, although at lower numbers than that exhibited during the embryo stages. The percentage of LSK cells in the adult liver was approximately 0.797% ± 0.105% of the Lin^−^ cells in the liver (Figure 1(c)), which was lower than that observed in the bone marrow (Figures 1(c) and 1(d)).

To further verify the hematopoietic activity of the LSK cells derived from the adult livers, we first tested the colony-formation activity of the liver mononuclear cells compared with the bone marrow mononuclear cells as a control using a methylcellulose semisolid medium assay. As expected, liver mononuclear cells could form GM-CFU and M-CFU clones (Figure 1(e)). As shown in Figure 1(f), 2.333 ± 0.577 GM-CFU clones and 2.667 ± 0.577 M-CFU clones from the liver mononuclear cells were detected on day 12 of culture. These results indicate that liver mononuclear cells contain hematopoietic progenitor cells and have the ability to form colonies, although this ability was weaker than that derived from the bone marrow. We next detected the colony-formation ability of LSK cells sorted from the liver and bone marrow by flow cytometry. We found that the LSK cells derived from the liver had the ability to form GM-CFU and M-CFU clones (Figure 1(g)); however, the total numbers of GM-CFU and M-CFU clones derived from the liver LSK cells were significantly lower than those isolated from the bone marrow (Figure 1(h)). These findings indicate that liver LSK cells can form hematopoietic clones, albeit to a weaker extent than bone marrow LSK cells. In summary, these results suggest the presence of hematopoietic progenitor cells in the adult murine liver and the hematopoietic potential of liver LSK cells.

### 3.2. Murine Adult Liver LSK Cells Are Capable of Generating Both Lymphoid and Myeloid Cells *In Vivo*

**Figure 2:**
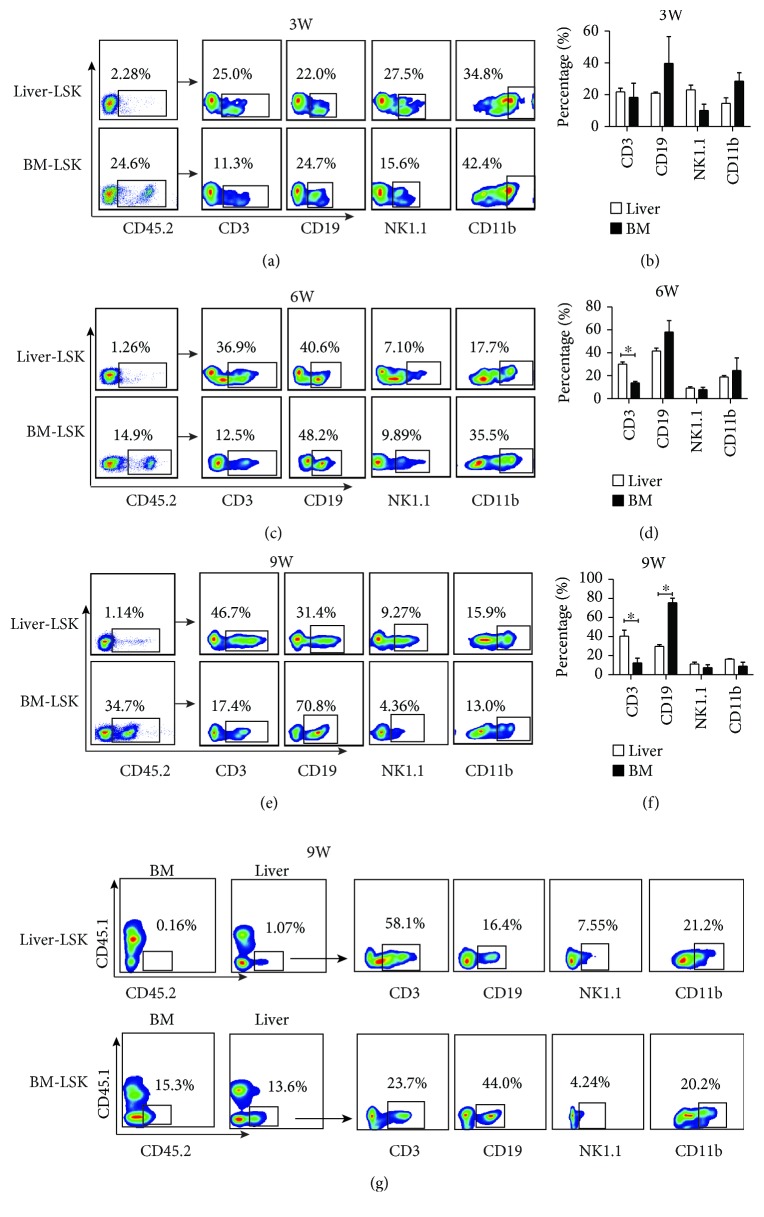
The ability of adult liver LSK cells to differentiate into lymphocytes and myeloid cells in vivo. (a, c, e) 2 × 10^4^ liver or BM LSK cells obtained from CD45.2 mice were mixed with 2 × 10^5^ unfractionated CD45.1^+^ competitor bone marrow cells and intravenously injected into lethally irradiated CD45.1 recipient mice. Peripheral blood was collected weekly. The proportion of differentiated lymphoid (CD3^+^, CD19^+^, and NK1.1^+^) and myeloid (CD11b^+^) lineages of CD45.2^+^ cells in the peripheral blood of CD45.1^+^ recipient mice was detected by FACS at weeks 3, 6, and 9. The experiments were repeated three times. (b, d, f) Statistical chart of the proportion of CD3^+^ T, CD19^+^ B, NK1.1^+^ NK, and CD11b^+^ myeloid cells derived from CD45.2^+^ donor cells in the peripheral blood of CD45.1^+^ recipient mice at weeks 3, 6, and 9 (*n* = 3). Bars represent the mean ± SEM of three independent experiments. ^∗^ *P* < 0.05. (g) Flow cytometric plots show the percentages of CD45.2^+^ cells in the liver of CD45.1^+^ recipient mice detected by FACS at the 9^th^ week. The proportions of differentiated lymphoid (CD3^+^, CD19^+^, and NK1.1^+^) and myeloid (CD11b^+^) lineages of CD45.2^+^ cells in the liver of CD45.1^+^ recipient mice were detected by FACS.

As early as 1996, Taniguchi et al. found that LSK cells in the adult liver could reconstruct the multilineage hematopoiesis [3]. However, the proportion of CD3^+^ T cells and NK1.1^+^ cells was not detected in prior reports. To confirm the hematopoietic function of LSK cells in the murine adult liver, the repopulating capacity of LSK cells was examined with a competitive repopulating assay. A total of 2 × 10^4^ LSK cell suspensions obtained from either the liver or bone marrow of donor CD45.2^+^ mice was mixed with 2 × 10^5^ competitor bone marrow mononuclear cell suspensions from CD45.1 mice and injected into lethally irradiated CD45.1^+^ recipients to confirm the hematopoietic-reconstitution activity (Figure S1(a) and 1(b)). Donor-derived (CD45.2^+^) CD3^+^, CD19^+^, NK1.1^+^, and CD11b^+^ cells were detected in the peripheral blood of CD45.1^+^ recipient mice at different time points following transplantation. As shown in Figures 2(a)–2(f), the LSK cells derived from the liver can give rise to a high proportion of lymphocytes (including T and B cells) from three weeks following transplantation; however, the hematopoietic reconstitution ability of the LSK cells derived from the liver is significantly weaker than that isolated from the bone marrow. We found that LSK cells from the liver preferentially differentiate into T cells compared with those from the bone marrow. Moreover, LSK cells derived from the bone marrow mainly produced B cells and generated fewer T cells. There were no differences observed regarding the repopulation of NK and myeloid cells between the liver- and bone marrow-derived LSK cells (Figures 2(a)–2(f)). To determine the destination and sites of differentiation of liver LSK cells after transplantation, we detected CD45.2^+^ cells in the recipient liver and BM after LSK transplantation at the ninth week. The results showed that CD45.2^+^ cells can be detected in the liver but not in the BM in mice receiving liver LSK transplantation (Figure 2(g)). Similar with those in the peripheral blood (Figure 2(e)), differentiated CD3^+^ T cells, CD19^+^ B cells, NK1.1^+^ T cells, and CD11b^+^ myeloid cells, especially CD3^+^ T cells, from CD45.2^+^ donor cells were detected in the liver (Figure 2(g)). However, for BM LSK transplantation, CD45.2^+^ cells were detected in both recipient BM and liver; CD45.2^+^ donor-derived CD3^+^ T cells, CD19^+^ B cells, NK1.1^+^ T cells, and CD11b^+^ myeloid cells were generated in the liver, among which CD19^+^ B cells were dominant (Figure 2(g)). These results suggested that liver-derived LSK cells specifically home to the liver, where they further differentiate into lymphocytes and myeloid cells, but rarely return to the BM. However, BM-derived LSK cells can move to both the BM and liver. Taken together, these results indicated that adult liver HSPCs can destine to the liver where they differentiate into both lymphoid and myeloid cells, particularly with the preferential T cell differentiation.

### 3.3. Kupffer Cells Promote LPS-Induced Liver Hematopoiesis

**Figure 3:**
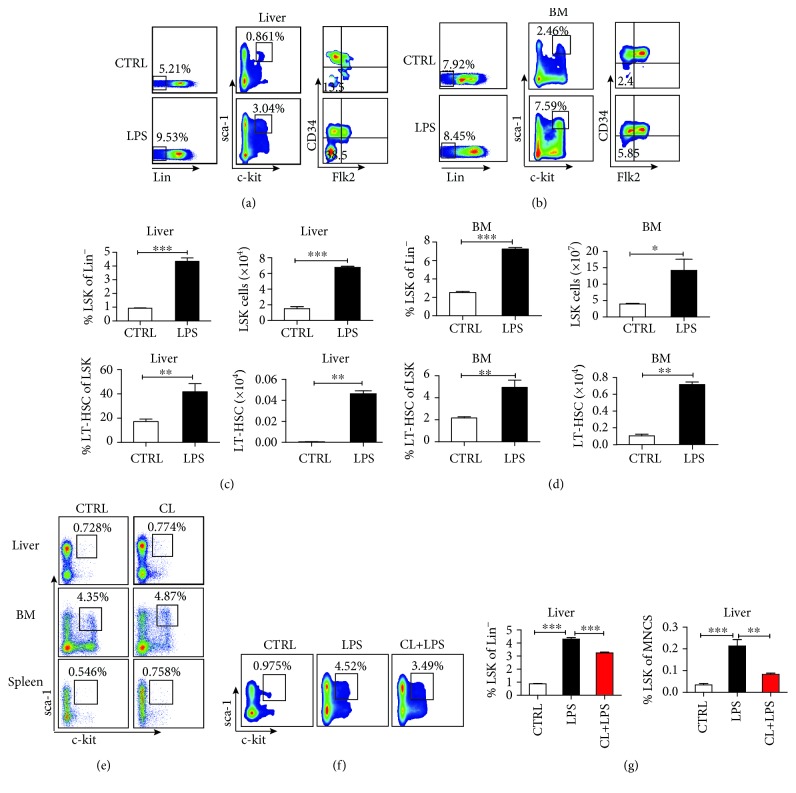
Kupffer cells promote liver extramedullary hematopoiesis. (a, b) Flow cytometric plots show the proportion of LSKs in Lin^−^ cells and LT-HSCs (LSK Flk2^−^ CD34^−^) in LSKs from the BM or liver of PBS or LPS-treated mice (*n* = 3‐5). Mice were intraperitoneally injected with LPS (10 *μ*g/mL) or PBS for three days. (c, d) The statistical percentage of LSKs in Lin^−^ cells and LT-HSCs in LSK cells from the liver or BM of mice treated with PBS or LPS (left). Statistical analysis of the absolute number of liver or BM LSK and LT-HSC cells (right). (e) Flow cytometric plots show the proportion of LSKs in Lin^−^ cells in the liver, BM, and spleen from the PBS or CL-treated groups. (f) Proportion of liver LSKs in the control group, LPS-treated group, and kupffer cell-depleted LPS-induced liver extramedullary hematopoiesis group (CL+LPS). (g) The statistical picture of the ratio of liver LSK cells to Lin^−^ or mononuclear cells from the PBS, LPS, and LPS+CL groups. The data represent three independent experiments with 3-5 mice per group. Bars represent mean ± SEM. ^∗^ *P* < 0.05; ^∗∗^ *P* < 0.01; ^∗∗∗^ *P* < 0.001.

Next, we explored whether the adult liver, like the hematopoietic niche in the bone marrow, contains factors that regulate the retention, proliferation, quiescence, and differentiation of HSPCs. Multiple cellular and molecular components are involved in the maintenance of the bone marrow HSC niche. In particular, it has been reported that macrophages contribute to HSPC maintenance in the bone marrow by CXCL12-CXCR4 chemokine signaling [10, 27–31]. Thus, we investigated whether kupffer cells, as the important resident macrophages of the liver, participate in the maintenance and promotion of the HSPC niche in the liver and the associated mechanism. Since there is only a small number of hematopoietic stem cells located in the normal adult liver during a quiescent state, we used a LPS-stimulated murine extramedullary hematopoiesis model [1] to promote adult liver hematopoiesis. We detected the frequency of liver LSK cells following an intraperitoneal injection of LPS (Figure S2). Firstly, we measured the number of the LSK cells and long-term hematopoietic stem cells (LT-HSC, Lin^−^Sca-1^+^ckit^+^ Flk2^−^CD34^−^) from the bone marrow and liver by flow cytometry. The unstimulated mouse liver contained only a few LSK (approximately 0.925% ± 0.067% of Lin^−^ cells) and LT-HSC (approximately 15.35% ± 2.032% of LSK cells) cells. However, the absolute numbers of liver LSK and LT-HSC cells were dramatically increased in LPS-treated mice compared with unstimulated mice (LSK: from 1.500 × 10^4^ ± 0.458 × 10^4^ increased to 6.767 × 10^4^ ± 0.231 × 10^4^; LT-HSC: from 0.005 × 10^4^ ± 0.002 × 10^4^ increased to 0.046 × 10^4^ ± 0.049 × 10^4^) (Figures 3(a) and 3(c)). Similar to the liver, bone marrow LSK cells and LT-HSCs in LPS-treated mice also increased (Figures 3(b) and 3(d)). These results suggest that LPS stimulation promotes extramedullary liver hematopoiesis, similar to the effect on spleen hematopoiesis [1].

To investigate the contribution of kupffer cells in the process of liver extramedullary hematopoiesis, we intravenously injected CL into LPS-treated mice to delete macrophages and kupffer cells (Figure S2). We first observed that the treatment with CL alone did not affect the distribution of HSPCs in the BM, liver, and spleen (Figure 3(e)). As shown in Figure S3, treatment with CL significantly reduced the proportion of kupffer cells (CD11b^+^ F4/80^+^) in the liver, and the percentage of hepatic LSK cells in LPS-treated mice was also significantly reduced (Figures 3(f) and 3(g)). Based on the above data, we propose that the kupffer cells may contribute to LPS-induced liver hematopoiesis.

### 3.4. Kupffer Cells Sustain Liver HSPCs to Differentiate into T and B Cells *In Vitro*

**Figure 4:**
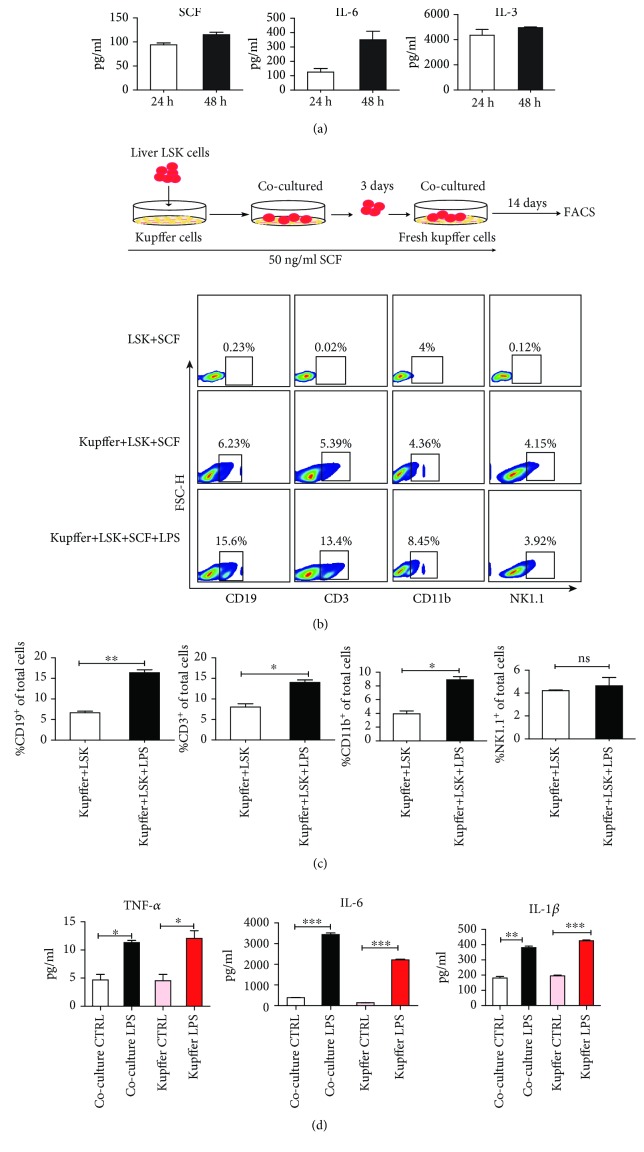
Kupffer cells secrete hematopoiesis-promoting cytokines and promote the differentiation of liver HSPCs. (a) Collagenase IV was used to digest the liver tissue, and kupffer cells were isolated by density gradient centrifugation. The cells were then inoculated into 24-well plates (1 × 10^5^ cells/well). Kupffer cells were further purified by cell adhesion selection. After culturing for 30 min, the supernatant was absorbed and washed three times with 1× PBS, then replaced with fresh DMEM medium (500 *μ*L). Cell culture supernatants were collected at 24 h and 48 h. An ELISA was used to detect the levels of SCF, IL-6, and IL-3 in the kupffer cell culture supernatants. The bars represent the mean ± SEM of three independent experiments. (b) The sorted liver LSK cells were seeded onto kupffer cell monolayers in the presence of SCF (50 ng/mL), and fresh kupffer cells were replaced every three days. The cocultured cells were collected and analyzed by flow cytometry on days 7 and 14. The proportion of lymphocytes (CD3^+^ T, CD19^+^ B, and NK1.1^+^ NK cells) and myeloid cells (CD11b^+^ cells) gated from total cells in the coculture system was detected by flow cytometry on day 14. (c) Statistical chart of the proportion of CD3^+^, CD19^+^, NK1.1^+^, and CD11b^+^ cells among the total coculture cells in the kupffer+LSK+SCF and kupffer+LSK+SCF+LPS coculture groups. (d) The level of proinflammatory factor IL-6, TNF-*α*, and IL-1*β* in the coculture supernatants from kupffer+LSK+SCF, kupffer+LSK+SCF+LPS, kupffer+CTRL, and kupffer+LPS groups was detected by ELISA. The data are represented as the mean ± SEM. ns: not significantly different. ^∗^ *P* < 0.05; ^∗∗^ *P* < 0.01.

To explore how kupffer cells contribute to liver hematopoiesis, we assessed the level of hematopoietic growth factors (SCF, IL-6, and IL-3) [32] secreted by kupffer cells by culturing freshly isolated kupffer cells for 24 h and 48 h *in vitro*. As shown in Figure 4(a), certain levels of SCF, IL-6, and IL-3 can be detected in the supernatants of cultured kupffer cells, suggesting that kupffer cells constitutively express these three hematopoietic growth factors.

Next, we evaluated whether kupffer cells could support the proliferation and differentiation of liver HSPCs. We cocultured liver LSK cells and freshly isolated kupffer cells in the presence of SCF with or without 1 *μ*g/mL LPS for 14 days and examined the proportion of differentiated lymphoid and myeloid cells at various time points. On the seventh day, a low percentage of CD3^+^ T, CD19^+^ B, NK1.1^+^, and CD11b^+^ cells was detected in the coculture system (data not shown). On day 14, we observed that liver LSK cells cultured only with medium and SCF were unable to differentiate into lymphoid and myeloid cells. While the presence of kupffer cells increased the proportion of differentiated CD3^+^ T, CD19^+^ B cells in total cells of coculture system compared with the control group and adding LPS in the system enhanced this effect of kupffer cells (Figures 4(b) and 4(c)). To further study which factors promote the differentiation of liver HSPC after LPS stimulation, we assessed the level of proinflammatory factors in the coculture supernatants after LPS stimulation by ELISA. We found that LPS stimulation increased the levels of IL-6, TNF-*α*, and IL-1*β* in the coculture system (Figure 4(d)), while other cytokines such as IL-18 showed no significant difference. These results suggest that kupffer cells sustain the differentiation of liver LSK cells *in vitro* and LPS can promote this effect, during which some cytokines, such as IL-6, may play some regulatory role.

### 3.5. Kupffer Cells Promote the Proliferation of Liver HSPCs

**Figure 5:**
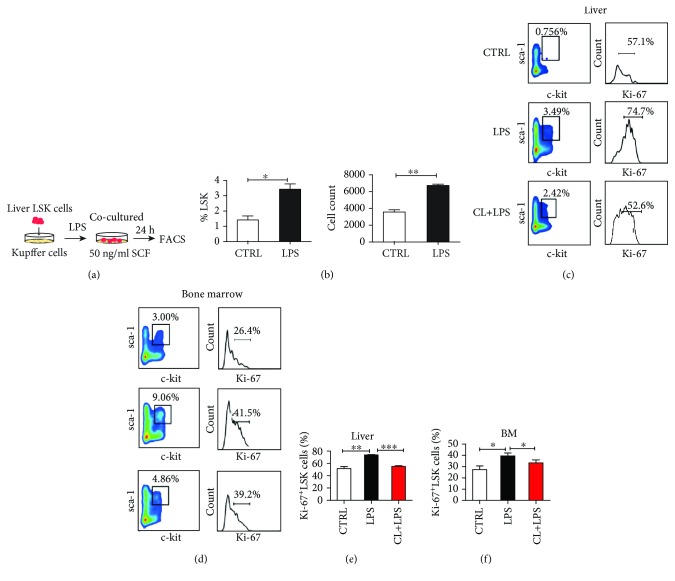
Kupffer cells can promote liver LSK cell proliferation. (a) A schematic map of the kupffer and LSK cell coculture. Freshly isolated kupffer cells were stimulated with 1 mg/mL LPS for 6 h, then cocultured with freshly sorted LSK cells for 24 h. (b) Statistical analysis of the percentage and absolute number of LSK cells among the mononuclear cells in the coculture system with or without LPS treatment. (c–f) An LPS-induced liver extramedullary hematopoiesis model was established with or without kupffer cell depletion. The proliferation of LSK cells in the liver and bone marrow was determined by Ki-67 labeling in the different treatment groups by flow cytometry. Mean ± SEMs from three independent experiments are presented (*n* = 3‐5). ^∗^ *P* < 0.05; ^∗∗^ *P* < 0.01; ^∗∗∗^ *P* < 0.001.

We further investigated the effect of kupffer cells on the proliferation of hepatic HSPCs. Kupffer cells were stimulated with 1 *μ*g/mL LPS for 6 h, after which the LSK cells were added to the kupffer cell monolayers and cocultured for 24 h. The proportion of LSK cells was measured (Figure 5(a)) and the results showed that the proportion and number of LSK cells significantly increased after coculturing with LPS-stimulated kupffer cells (Figure 5(b)). To explore how LPS-stimulated kupffer cells increased the proliferation of LSK cells, we assessed the proliferative capacity of LSK cells by detecting the expression of ki-67. First, we observed the proportion of ki-67^+^ LSK cells in the liver following an intraperitoneal injection of LPS and characterized the influence of kupffer cell depletion *in vivo* (Figure S2). We found that LPS stimulation augmented the percentage of hepatic ki-67^+^ LSK cells compared with the PBS-treated group (Figures 5(c) and 5(e)), while the removal of kupffer cells significantly reduced the proportion of hepatic ki-67^+^ LSK cells (Figures 5(c) and 5(e)). Similar results were observed in the bone marrow (Figures 5(d) and 5(f)). The above results indicate that kupffer cells indeed contribute to LPS-induced proliferation of HSPCs in the liver hematopoietic niche.

### 3.6. Kupffer Cells Promote LPS-Induced Liver Hematopoiesis and Lymphocyte Differentiation via ICAM-1 and LFA-1 Interaction

**Figure 6:**
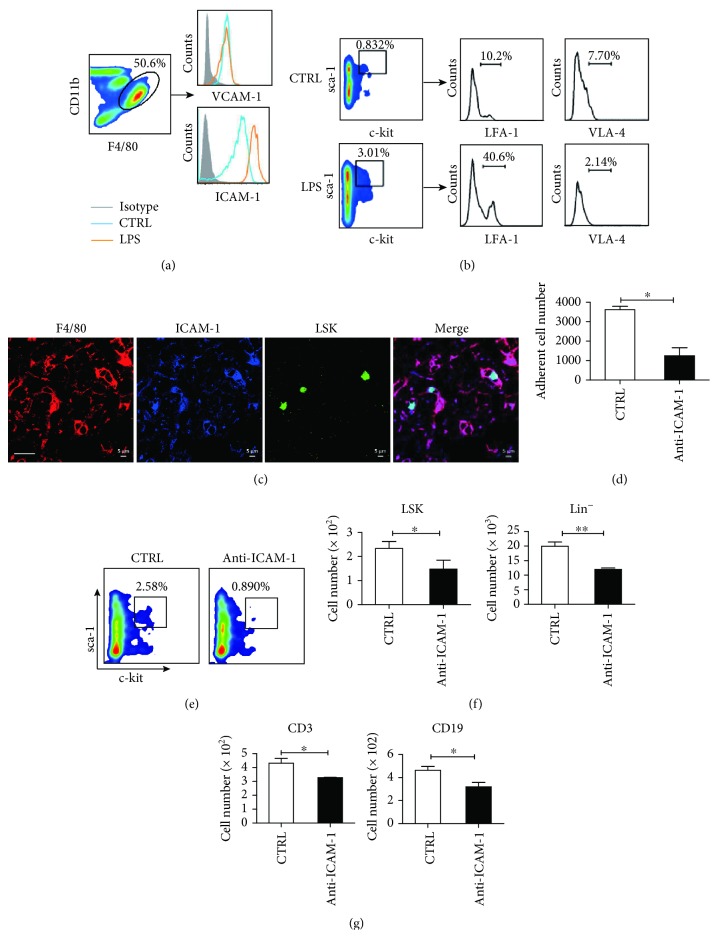
Kupffer cells promote liver hematopoiesis via the interaction between ICAM-1 and LFA-1. (a) The expression of ICAM-1 and VCAM-1 on kupffer cells from PBS- (blue) or LPS-treated mice (orange) was analyzed by flow cytometry. (b) Flow cytometry was used to measure the expression of LFA-1 and VLA-4 on liver LSK cells from PBS- or LPS-treated mice. (c) Immunofluorescence staining shows CFSE-labeled LSK cells (green) in close contact with ICAM-1^+^ (blue) kupffer cells (F4/80^+^, red) in the liver. Bar: 20 *μ*m. (d) Freshly isolated kupffer cells were preincubated with anti-ICAM-1 (10 *μ*g/mL) and subsequently cocultured with sorted liver CFSE-labeled LSK cells in the presence of anti-ICAM-1 for 24 h. Nonadherent CFSE^+^ cells were counted by flow cytometry and compared with those from cocultures in the absence of anti-ICAM-1. (e) 3.75 × 10^5^ Lin^−^ cells isolated from the liver were cocultured with freshly isolated kupffer cells in the presence of anti-ICAM-1 in vitro. The proportion of LSKs in the coculture system was detected by flow cytometry on day 7. (f) Statistical analysis of the absolute number of LSK cells and Lin^−^ cells. (g) Freshly isolated LSK cells were cocultured with kupffer cells in the presence of PBS or anti-ICAM-1. The cocultured cells were collected every seven days for analysis by flow cytometry. The statistical percentage of lymphocytes CD3^+^ T and CD19^+^ B cells in the LSK-kupffer cell coculture system. Three replicate wells were established for each group. Data are represented as the mean ± SEM. ^∗^ *P* < 0.05; ^∗∗^ *P* < 0.01.

The above results confirm that kupffer cells could promote the proliferation and differentiation of liver LSK cells. We next aimed to explore whether kupffer cells influence the maintenance of LSK cells in the liver. We measured the level of CXCL12, vascular cell adhesion molecule-1 (VCAM-1), intercellular cell adhesion molecule-1 (ICAM-1), c-kit ligand, and angiopoietin-1 expression in the liver, as these factors have been reported to be involved in the retention of HSPCs in the bone marrow niche [27, 29, 33–35]. We first detected the level of mRNA expression of these factors in the liver tissues isolated from the mouse model described in Figure S2. As shown in Figure S4(a), LPS stimulation markedly enhanced the levels of ICAM-1, VCAM-1, and CXCL12 mRNA in the whole liver tissues but did not influence the levels of c-kit ligand and angiopoietin-1. Depletion of kupffer cells significantly reduced the levels of ICAM-1 and VCAM-1 mRNA, but not CXCL12. We suspect that VCAM-1, ICAM-1, and CXCL12 expression on kupffer cells might contribute to the retention of HSPCs associated with LPS-induced liver extramedullary hematopoiesis. Therefore, we detected the level of CXCL12 in the liver tissue homogenates with an ELISA. We found that the changes in CXCL12 production were not statistically significant following kupffer cell depletion (Figure S4(b)). We then isolated kupffer cells and detected the changes in ICAM-1 and VCAM-1 expression by flow cytometry. Following LPS treatment, while the expression of ICAM-1 increased significantly, the expression of VCAM-1 did not change (Figure 6(a)). We further examined the expression of LFA-1 and VLA-4, which are the corresponding ligands for ICAM-1 and VCAM-1, respectively, on LSK cells. As shown in Figure 6(b), the expression of LFA-1, but not VLA-1, was substantially elevated following LPS treatment. According to these results, we speculate that the interaction between ICAM-1 and LFA-1 plays a major role in kupffer cell maintenance of HSPCs in the liver.

Next, we further tested whether liver LSKs reside closely to ICAM-1-expressing kupffer cells in the anatomical structure of the liver. For this assay, liver LSKs were sorted from CD45.2^+^ mice by flow cytometry, labelled with CFSE, and then intravenously transferred into CD45.1^+^ mice. Liver tissue sections were created after one day, and the location of LSK cells was observed using an immunofluorescence assay. We found that CFSE-labeled LSK cells were localized near ICAM-1^+^ kupffer cells in the liver (Figure 6(c)). To confirm the interaction between ICAM-1 and LFA-1, an adherent assay was performed. Freshly isolated kupffer cells were preincubated with an anti-ICAM-1 Ab (10 *μ*g/mL) and then cocultured with freshly sorted LSK cells. Nonadherent cells were counted after 12 h. We found that the level of adhesion between the LSK and kupffer cells decreased significantly after blocking ICAM-1 (Figure 6(d)). These results suggest that kupffer cells may retain small amounts of LSK cells in the hepatic sinusoid through the interaction between ICAM-1 and LFA-1.

We further examined the effect of kupffer cells on hematopoietic stem cells after blocking ICAM-1 in a coculture system. The liver Lin^−^ cells were sorted and cocultured with kupffer cells in the presence of SCF with or without an anti-ICAM-1 antibody for seven days, and the number of LSK cells was subsequently detected (Figure S4(c)). We found that the total number of LSK cells and Lin^−^ cells significantly declined following the ICAM-1 blockade (Figures 6(e) and 6(f)). We further analyzed the effect of kupffer cells on the differentiation of hematopoietic stem cells. Liver LSK cells were purified and cocultured with kupffer cells for 14 days, and then, the differentiated lymphoid and myeloid cells were detected. We observed that the total number of CD3^+^ T cells and CD19^+^ B cells declined when the anti-ICAM-1 treatment group was compared with the control group (Figure 6(g)). Taken together, these phenomena suggest that ICAM-1/LFA-1 plays a major role in the maintenance and differentiation of hepatic HPSCs by mediating close contact between kupffer cells and HSPCs.

## 4. Discussion

Previous reports have indicated that the adult liver contains HSPCs which possess hematopoietic-reconstitution ability [3–6]. However, no studies have comprehensively compared the differences in hematopoiesis and lymphogenesis between the adult liver and bone marrow. Moreover, the key factors in the adult liver microenvironment that contribute to liver hematopoiesis remain unclear. In this study, we confirmed that adult murine liver HSPCs differentiate into lymphoid and myeloid cells. Notably, the ability of liver LSK cells to generate T cells was significantly stronger than that of BM-LSK cells, whereas the BM-derived LSK cells primarily produced B cells. These findings suggest that liver hematopoiesis exhibits features that differ from bone marrow hematopoiesis. Furthermore, we confirmed that the liver resident macrophages, kupffer cells, can promote liver HSPCs to generate a high proportion of T and B lymphocytes through an interaction between ICAM-1 and LFA-1. Blocking ICAM-1 on kupffer cells impaired the adhesion, expansion, and differentiation of adult liver HSPCs. Our findings suggest a critical role of kupffer cells in the maintenance and promotion of adult mouse liver hematopoiesis, particularly T and B cell differentiation in the liver.

The hematopoietic microenvironment, particularly the stem cell niche, is critical for supporting the self-renewal, expansion, and differentiation of HSPCs in hemopoietic tissues [36]. In addition to the required growth factors (e.g., stem cell factors, flt3 ligand, IL- 6, and IL-3), direct interactions between HSPCs and cellular components (e.g., blood vessel endothelial cells, osteoblasts, and mesenchymal stem cells) within the stem cell niche are crucial for the regulation of hematopoiesis [11, 37, 38]. Although the cellular basis of the hematopoietic niche is clear in the bone marrow, the constituents and mechanism of the adult liver hematopoietic niche remain unclarified. Cardier and Barbera-Guillem reported that liver sinusoidal endothelial cells (LSECs) play a key role in supporting the proliferation and differentiation of HSPCs [39]. Moreover, evidence indicates that LSECs provide the signals required for the migration and homing of extramedullary hematopoietic stem cells and promote B lymphopoiesis [40, 41]. However, the role of kupffer cells in liver hematopoiesis has not been fully clarified. Otsuka et al. reported that kupffer cells act as stromal cells and support extramedullary erythropoiesis in the livers of splenectomized mice [42]. Moreover, in a phenylhydrazine-induced extramedullary hematopoiesis model, F4/80^+^ macrophages were found to be tightly surrounded by erythroblasts in the liver sinusoids, similar to erythropoiesis in the fetal liver [43]. However, the effects and molecular mechanism of kupffer cells on lymphocytopoiesis are poorly understood. In the present study, we found that kupffer cells promote liver hematopoiesis. Our data demonstrates that kupffer cells both contribute to supporting the proliferation of liver HSPCs and sustain liver HSPCs, which differentiate into lymphocytes. Regarding the associated mechanism, we propose that (1) under steady-state conditions, kupffer cells secrete the hematopoietic-promoting factors IL-3, IL-6, and SCF, which support the maintenance of HSPCs and (2) kupffer cells promote liver hematopoiesis and lymphogenesis via an ICAM-1 and LFA-1 interaction. To our knowledge, this is the first report to investigate the role and mechanisms of kupffer cells in the promotion of lymphogenesis during liver extramedullary hematopoiesis. Our data also provides evidence that kupffer cells are an important component of the liver hematopoietic niche.

Although some evidence suggests that there may be different characteristics between liver and bone marrow hematopoiesis, there is a lack of comprehensive comparative studies. Golden-Mason et al. [8] have reported that the normal adult human liver is capable of supporting T cell development. They found that the normal adult human liver contains a considerable proportion of HSCs expressing lymphoid-associated markers, whereas the majority of CD34^+^ cells in the bone marrow express the myeloid-associated antigen, CD33, and B cell marker, CD19^+^, but exhibit fewer T cell progenitors [8]. Based on the findings of a previous study [44], the present study compared the hematopoietic and lymphopoietic capacity between bone marrow- and liver-derived HSPCs using both an *in vivo* model and an *in vitro* coculture. We observed that liver-derived LSK cells specifically home to the liver, where they further differentiate into lymphocytes and myeloid cells, then enter into the peripheral circulation. However, BM-derived LSK cells can move to both the BM and liver. It is notable that liver LSK cells preferentially differentiate into T cells, whereas LSK cells derived from the bone marrow mainly produced B cells and generated fewer T cells. There are no differences regarding the repopulation of NK and myeloid cells between the liver- and bone marrow-derived LSK cells. These findings are consistent with previous reported evidence regarding the supportive role of the human liver in T cell development [8]. Extrathymic T cell differentiation in the liver has also been observed in tumor-bearing mice and patients with tumors or undergoing a liver transplantation [8, 45–47]. It is suggested that T cells of extrathymic origin may be involved in tumor immunity and contribute to immune tolerance following organ transplantation [7, 48, 49]. We also found that LPS could help kupffer cells to promote the differentiation of liver HSPCs into T and B cells. Recent studies have highlighted the important role of LPS production in the pathogenesis of liver diseases, such as NAFLD/NASH and hepatocellular carcinoma (HCC) [50]. Our results may help to study liver hematopoiesis under the state of these liver diseases.

However, the origin of liver HSPCs and the function of T cells derived from the liver HSPCs are still unclear. Next, we need to further confirm whether adult liver HSPCs are derived from a remnant of fetal HSPCs or from bone marrow-circulating HSPCs. It is also necessary to illuminate the function and significance of liver extramedullary hematopoiesis, particularly regarding therapy for liver tumors and related diseases.

## 5. Conclusions

In summary, the findings of the present study demonstrate that adult murine liver HSPCs exhibit hematopoietic activity, primarily differentiating into T and B lymphocytes. We further confirmed that kupffer cells can promote the adhesion, proliferation, and differentiation of adult liver HSPCs by the interaction between ICAM-1 and LFA-1. These findings provide important insight into understanding the liver hematopoietic microenvironment and may aid in the development of novel therapies for liver-related diseases and the maintenance of immune tolerance following liver transplantation.

## Data Availability

The data used to support the findings of this study are included within the article.

## Supplementary Materials

Supplementary Materials Supplementary Table S1: mouse antibody list. Supplementary Table S2: primer sequences used for real-time PCR. Supplementary Figure S1: analysis and sorting strategy of the bone marrow, liver, and fetal liver LSK cells. Supplementary Figure S2: flow chart of kupffer cell depletion and LPS treatment. Supplementary Figure S3: clodronate-liposome treatment significantly reduced the proportion of kupffer cells in the liver. Supplementary Figure S4: factors promoting and maintaining liver hematopoiesis.
