# Supplementary material for: Kupffer Cells Promote the Differentiation of Adult Liver Hematopoietic Stem and Progenitor Cells into Lymphocytes via ICAM-1 and LFA-1 Interaction
Source: Stem Cells Int. 2019 Jul 1;2019:4848279. doi: 10.1155/2019/4848279 (PMC6636495; doi:10.1155/2019/4848279)
Supplement: Supplementary Materials — Supplementary Table S1: mouse antibody list. Supplementary Table S2: primer sequences used for real-time PCR. Supplementary Figure S1: analysis and sorting strategy of the bone marrow, liver, and fetal liver LSK cells. Supplementary Figure S2: flow chart of kupffer cell depletion and LPS treatment. Supplementary Figure S3: clodronate-liposome treatment significantly reduced the proportion of kupffer cells in the liver. Supplementary Figure S4: factors promoting and maintaining liver hematopoiesis. [file 4848279.f1.doc]

**Supplemental Tables**

**[Table S1](http://www.sciencedirect.com/science/article/pii/S0304383515006084?via=ihub#ec0010): Mouse antibody list**

| **Marker** | **Clone** | **Fluor** | **Isotype Control** | **Vendor** |
| --- | --- | --- | --- | --- |
| Lineage antibody | cat51-9006964 | percpcy^TM^5.5 | PerCP-Cy™5.5 Mouse Lineage Isotype Control Cocktail | BD |
| sca-1 | D7 | APC | [APC Rat IgG2a, κappa](https://www.biolegend.com/en-us/products/apc-rat-igg2a--kappa-isotype-ctrl-1838) | Biolegend |
| CD117 | 2B8 | PE-eFluor610 | PE-eFluor610 Rat IgG2b, kappa | ebioscience |
| Flk2 | A2F10 | PE | PE Rat IgG2a, kappa | ebioscience |
| CD34 | RAM34 | FITC | [FITC Rat IgG2a, κappa](http://www.bdbiosciences.com/cn/reagents/research/antibodies-buffers/immunology-reagents/anti-mouse-antibodies/cell-surface-antigens/fitc-rat-igg2a-isotype-control-r35-95/p/553929) | BD |
| ICAM-1 | YN1/1.7.4 | APC | [APC Rat IgG2b, κappa](https://www.biolegend.com/en-us/products/apc-rat-igg2b--kappa-isotype-ctrl-1851) | Biolegend |
| VCAM-1 | 429MVCAMA | FITC | [FITC Rat IgG2a, κappa](https://www.biolegend.com/en-us/products/fitc-rat-igg2a--kappa-isotype-ctrl-1841) | Biolegend |
| VLA-4 | R1-2 | FITC | [FITC Rat IgG2b, κappa](https://www.biolegend.com/en-us/products/fitc-rat-igg2b--kappa-isotype-ctrl-1854) | Biolegend |
| LFA-1 | H155-78 | PE-cy7 | [PE/Cy7 Rat IgG1, κappa](https://www.biolegend.com/en-us/products/pe-cy7-rat-igg1--kappa-isotype-ctrl-1934) | Biolegend |
| Ki-67 | 16A8 | PE | [PE Rat IgG2a, κappa](https://www.biolegend.com/en-us/products/pe-rat-igg2a--kappa-isotype-ctrl-1843) | Biolegend |
| CD11b | M1/70 | PE | [PE Rat IgG2b, κappa](https://www.biolegend.com/en-us/products/pe-rat-igg2b--kappa-isotype-ctrl-1856) | Biolegend |
| CD3 | 145-2C11 | PE-cy7 | [PE/Cy7 Armenian Hamster IgG](https://www.biolegend.com/en-us/products/pe-cy7-armenian-hamster-igg-isotype-ctrl-1920) | Biolegend |
| NK1.1 | PK136 | APC-CY7 | [APC/Cy7 Mouse IgG2a, κappa](https://www.biolegend.com/en-us/products/apc-cy7-mouse-igg2a--kappa-isotype-ctrl-1923) | Biolegend |
| NK1.1 | PK136 | APC | [APC Mouse IgG2a, κappa](https://www.biolegend.com/en-us/products/apc-mouse-igg2a--kappa-isotype-ctrl-1397) | Biolegend |
| CD45.2 | 104 | APC | APC Mouse IgG2a κappa | BD |
| CD45.1 | A20 | PE-CF594 | PE-CF594 Mouse IgG2a, κappa | BD |
| CD19 | 6D5 | PE | [PE Rat IgG2a, κappa](https://www.biolegend.com/en-us/products/pe-rat-igg2a--kappa-isotype-ctrl-1843) | Biolegend |
| CD19 | 6D5 | APC-CY7 | [APC/Cy7 Rat IgG2a, κappa](https://www.biolegend.com/en-us/products/apc-cy7-rat-igg2a--kappa-isotype-ctrl-2321) | Biolegend |
| F4/80 | PE/Dazzle™ 594 | PE-CF594 | [PE/Dazzle™ 594 Rat IgG2a, κappa](https://www.biolegend.com/en-us/products/pe-dazzle-594-rat-igg2a--kappa-isotype-ctrl-9828) | Biolegend |

**[Table S2](http://www.sciencedirect.com/science/article/pii/S0304383515006084?via=ihub#ec0010)**: **Primer sequences used for real-time PCR**

| **Names** | **Forward** | **Reverse** |
| --- | --- | --- |
| *Cxcl12* | CGCCAAGGTCGTCGCCG | TTGGCTCTGGCGATGTGGC |
| *Angpt1* | CTCGTCAGACATTCATCATCCAG | CACCTTCTTTAGTGCAAAGGCT |
| *Kitl* | CCCTGAAGACTCGGGCCTA | CAATTACAAGCGAAATGAGAGCC |
| *Vcam-1* | GACCTGTTCCAGCGAGGGTCTA | CTTCCATCCTCATAGCAATTAAGGTG |
| *Icam-1* | GTTCTCTAATGTCTCCGAGGC | CTTCAGAGGCAGGAAACAGG |
| *Gapdh* | TGTGTCCGTCGTGGATCTGA | CCTGCTTCACCACCTTCTTGA |

**Supplemental Figures**


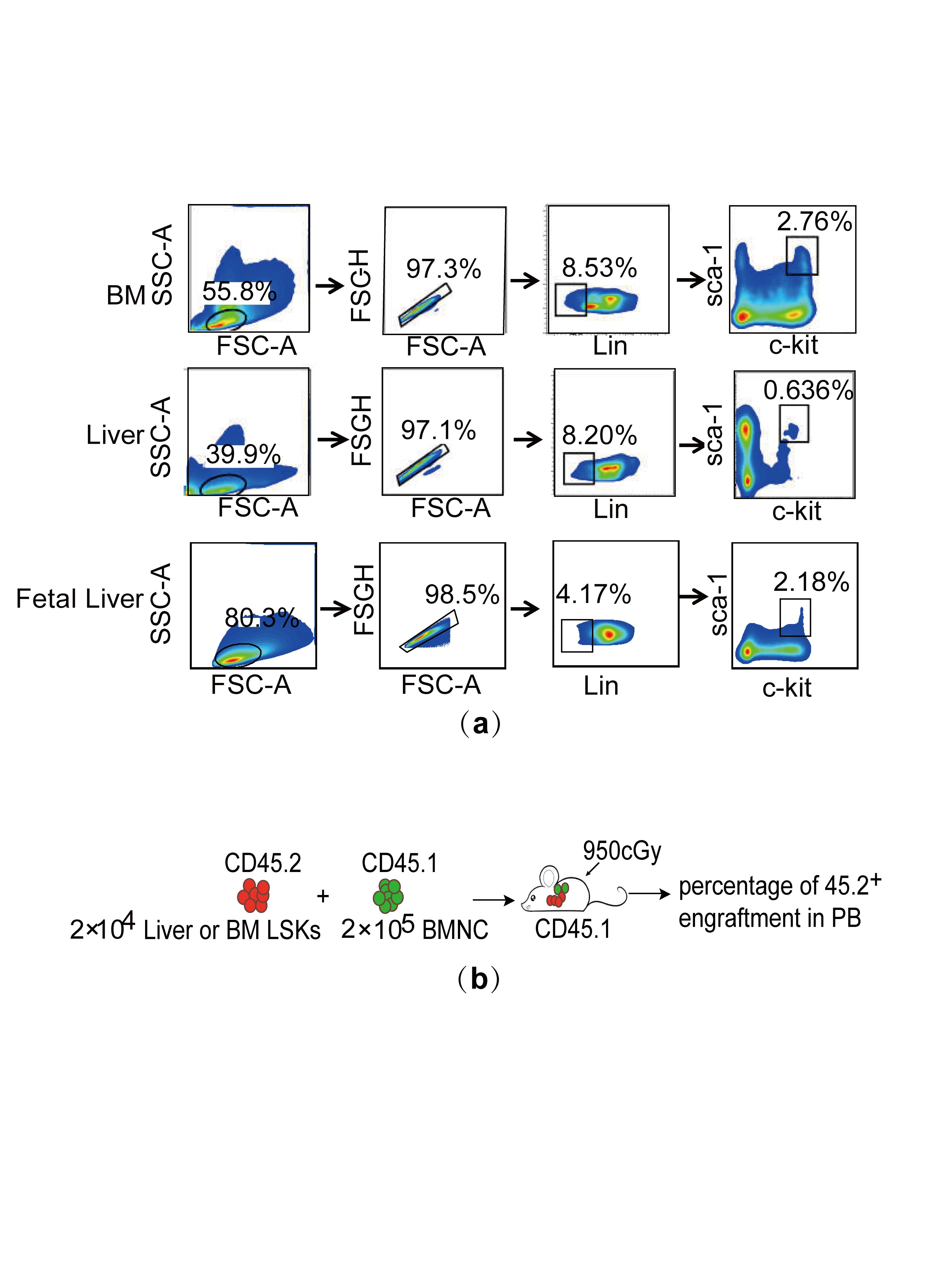


Figure S1. Analysis and sorting strategy of the bone marrow, liver and fetal liver LSK cells. (a) Lymphocytes were gated according to FSC-A/SSC-A, and the adherent cells were removed according to FSC-A/FSC-H. Next, the Lin^-^ cells were gated. The c-kit+ sca-1+ cells were further gated from the Lin^-^ cells. (b) Flow chart of transfer experiment. 2 × 10^4^ liver or BM LSK cells obtained from CD45.2 mice were mixed with 2 × 10^5^ unfractionated CD45.1^+^ competitor bone marrow cells, and intravenously injected into lethally irradiated CD45.1 recipient mice. Peripheral blood was collected weekly.


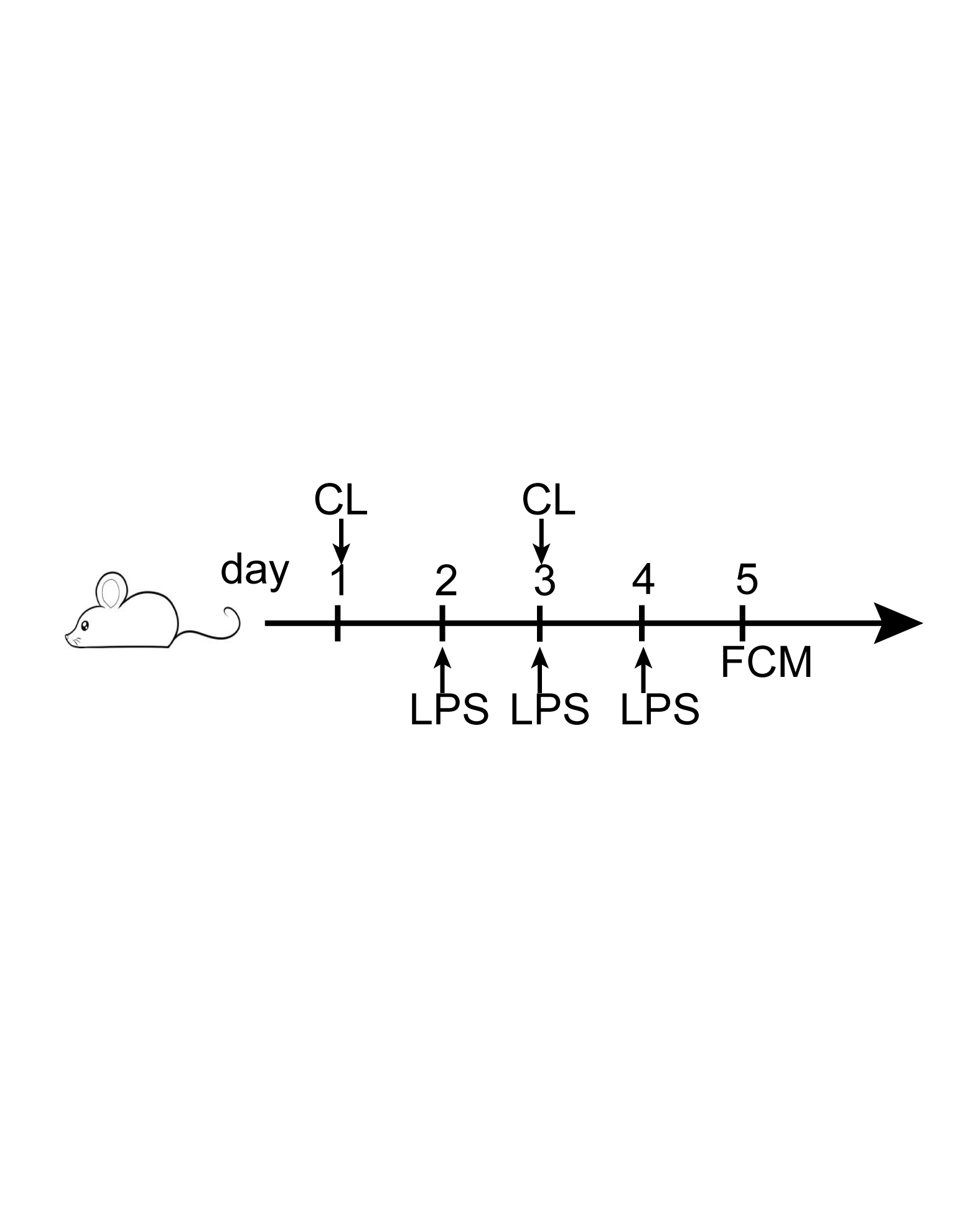


Figure S2. Flow chart of kupffer cell depletion and LPS treatment. An intraperitoneal injection of clodronate-liposome (CL) was administered to mice on the first and third day to deplete kupffer cells. A continuous injection of LPS (10 μg/mL) occurred for three days from day 2 to day 4. Flow cytometry was performed after five days.


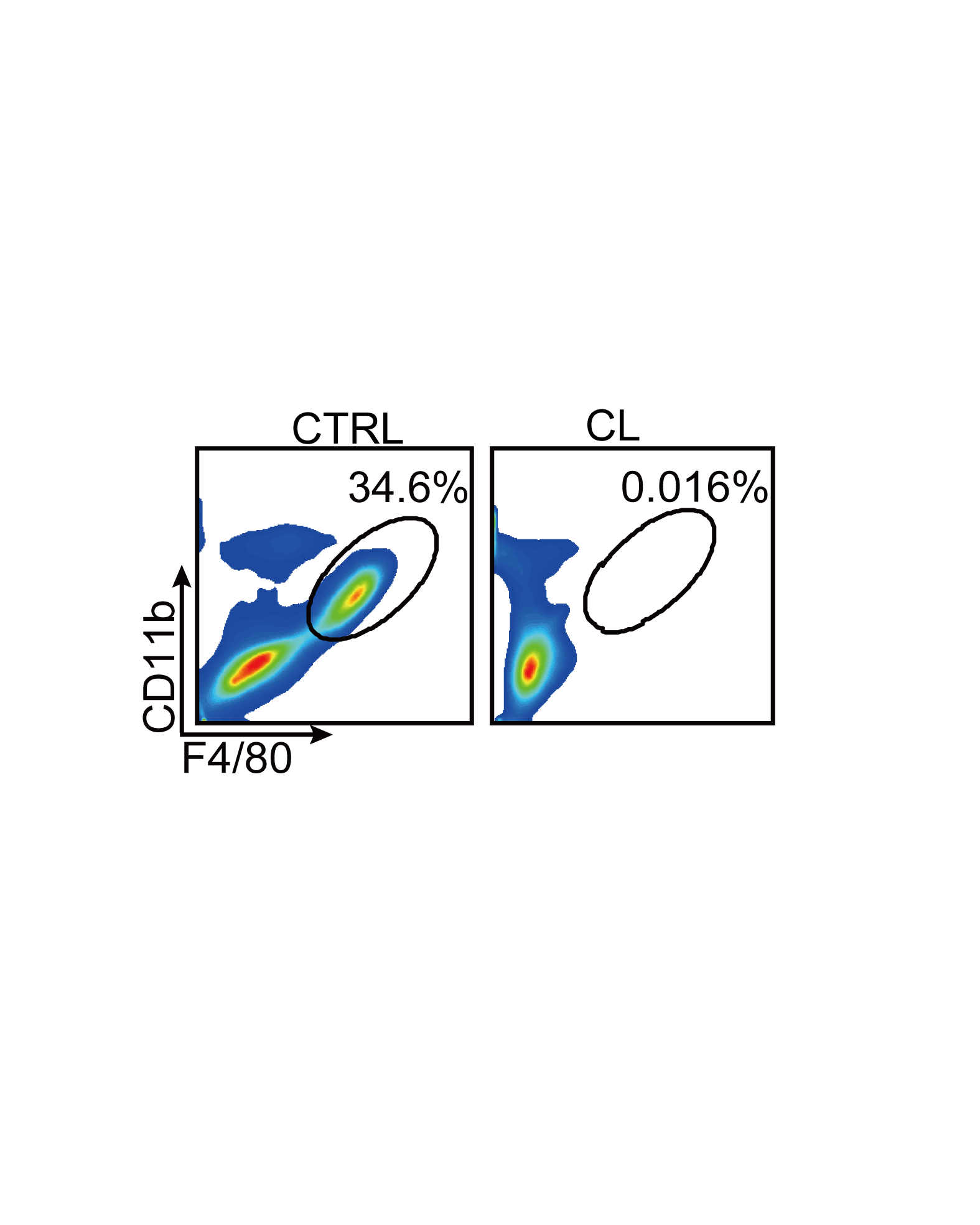


Figure S3. Clodronate-liposome treatment significantly reduced the proportion of kupffer cells in the liver. Plots show the percentage of liver kupffer cells following CL treatment. Mice were injected with CL via the abdominal cavity on day 1 and day 3, and the depletion effect was detected on day 5 by flow cytometry.


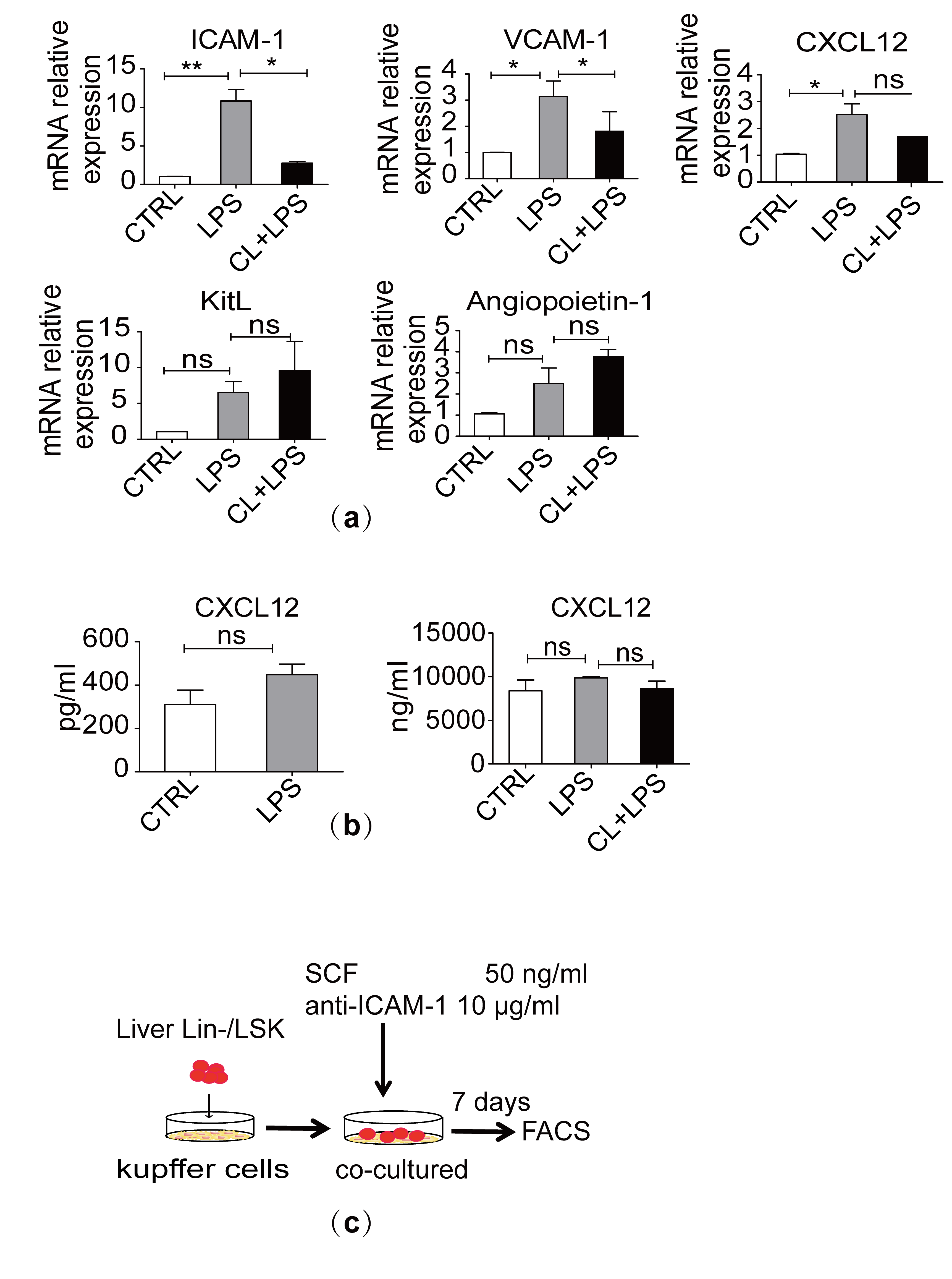


Figure S4. Factors promoting and maintaining liver hematopoiesis. (a) The level of hematopoiesis-related factor mRNA expression was detected by real-time PCR in the lysates of hepatic tissues from PBS (control), LPS, and LPS + CL treated mice (n = 5 – 6). (b) The level of CXCL12 in the supernatants of LPS (1 μg/mL) or PBS-treated kupffer cells for 6 h (left) and in the liver homogenates from PBS (control), LPS, and LPS + CL-treated mice (right) was detected with an ELISA. (c) A flow chart of co-culture experiments. Sorted LSK or Lin- cells were cocultured with freshly isolated kupffer cells in the presence of SCF (50 ng/mL) and anti-ICAM-1 (10 μg/mL), and the cells were collected for detection after seven days. The data are represented as the mean ± SEM. ns: not significantly different. *p < 0.05; **p < 0.01.
